# Improving Documentation in Plastic Surgery: Sister Bra Size Group and BMI Are More Indicative of Breast Weight Than Cup Size

**DOI:** 10.1093/asj/sjaf114

**Published:** 2025-06-23

**Authors:** Eliana Jolkovsky, Meghan N Miller, Ainaz Dory Barkhordarzadeh, Stacy Piva, Tahera Alnaseri, Ginger C Slack

## Abstract

**Background:**

Accurate documentation of breast size is critical for surgical planning, insurance authorization, and research in breast reconstruction. However, breast size is inconsistently recorded, often limited to brassiere cup size, which may not reliably predict breast tissue weight.

**Objectives:**

The authors of this study aim to evaluate whether “sister bra size group”—a previously unverified classification incorporating both bra cup and band sizes—better correlates with breast weight than cup size alone.

**Methods:**

A retrospective review was conducted of 209 patients (395 breasts) who underwent mastectomy between 2017 and 2023 at a single institution. Preoperative bra cup and band sizes, mastectomy specimen weights, BMI, and demographic characteristics were recorded. Patients were categorized into sister bra size groups. Spearman's correlation coefficients and multivariate linear regression were used to evaluate associations with breast weight.

**Results:**

Sister bra size group showed the strongest Spearman's correlation with breast weight (*ρ* = 0.76), followed by cup size (*ρ* = 0.67), BMI (*ρ* = 0.61), and band size (*ρ* = 0.48). Age did not have a significant correlation with mastectomy specimen weight (*ρ* = 0.02). In multivariate analysis, sister size (*P* = .016) and BMI (*P* < .001) remained statistically significant predictors of breast weight, whereas cup and band sizes did not.

**Conclusions:**

Cup size alone is not a reliable predictor of breast tissue weight. Sister bra size groups provide a stronger correlation and a more accurate alternative. Incorporating this variable into clinical documentation may improve preoperative planning and create a more standardized framework for research.

**Level of Evidence: 4 (Therapeutic):**

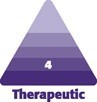

Electronic health record (EHR) systems have transformed the quality of patient care by capturing health information and facilitating the exchange of medical information.^1^ EHR systems have also improved large-scale clinical research by enabling easier collection and extraction of patient data.^2^ The accuracy of documentation is therefore critical. One area in plastic and reconstructive surgery in need of improvement and uniformity is the documentation of brassiere sizes, which is often limited to cup size. Breast cancer is currently the most diagnosed cancer around the world.^3^ With a growing incidence rate, the United States could see 364,000 breast cancer cases by 2040.^4^ A mastectomy, which surgically removes breast tissue, is often used for breast cancer prevention and treatment. Predicting the amount of tissue to be removed in a mastectomy is helpful for both surgery planning and patient expectations. However, the unclear link between tissue mass and bra size can lead to discrepancies that result in patient dissatisfaction.^5^ This disconnect similarly applies to the planning of other reconstructive breast surgeries such as reduction, and we posit that improved EHR documentation will help to bridge this gap. Surgeons often predict breast weight solely with bra cup size, yet there is no evidence that cup size without band size is a reliable indicator of breast weight. Our team hypothesizes that documentation of a bra size should include both the band and cup sizes rather than bra cup size alone. We test the “Sister Bra Size Theory,” which assigns a bra size group based on both band and cup size. Before this study, the theory did not have any supporting data, yet it had been put forth by various online blogs and brassiere companies.^6,7^ It assumes that a bra with a smaller band size and larger cup size will similarly fit the wearer as a bra with a larger band size and smaller cup size would, and vice versa. For example, 34A and 32B will fit a wearer the same, the theory suggests. We hope that the results of our retrospective study of 209 mastectomy patients will assist both patients and surgeons in breast surgery planning and bridge a gap in communication between patient and provider. Improved documentation regarding breast size will have various additional implications, such as in completing health insurance claims and in providing more reliable and valid data for future breast reconstruction research.^8^

## METHODS

### Study Enrollment

**Figure 1.**
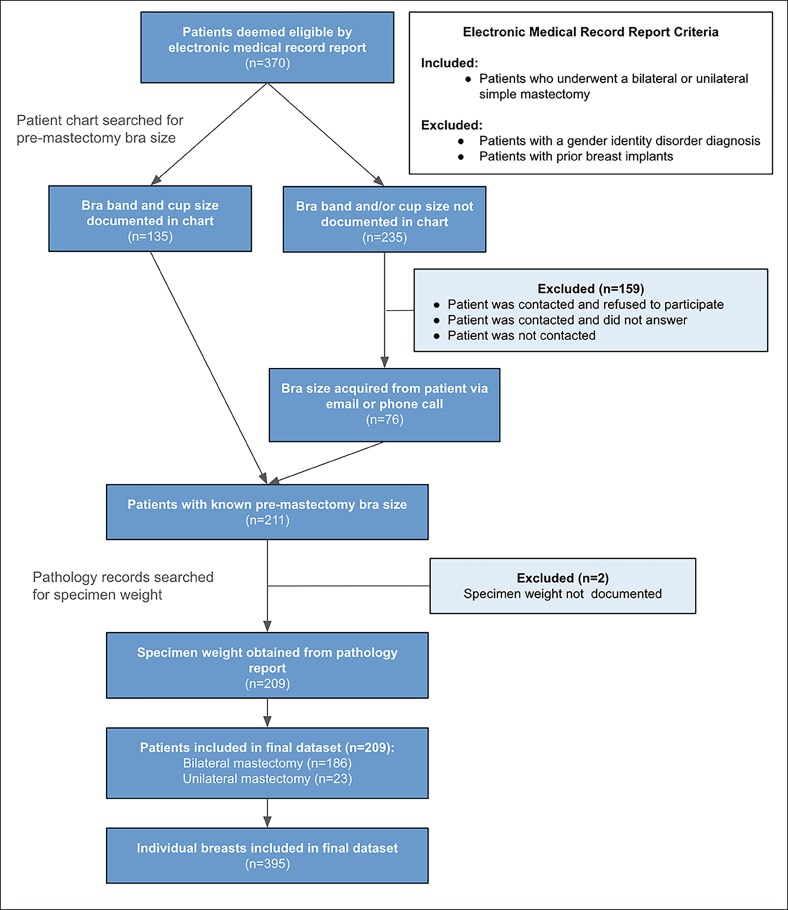
Study enrollment flow chart. Flow chart illustrating patient inclusion and exclusion criteria used to arrive at the final study population (*n* = 209 patients; *n* = 395 individual breasts).

This retrospective, single-site study was approved by the IRB of the UCLA Health System. Eligible patients were identified using a report generated by the UCLA Clinical and Translational Science Institute from the EHR system. The report included 370 patients who underwent unilateral or bilateral mastectomy at UCLA from December 1, 2017, to December 1, 2023, excluding patients with previous breast implants or a gender identity disorder diagnosis (Figure 1). The mastectomies in our study were performed by 8 different surgeons.

### Data Acquisition

Chart review was performed for all 370 patients to search for documentation of premastectomy bra size. A total of 135 patients had both band and cup sizes documented in the consult or preoperative visit note. The remaining 235 patients had incomplete or no documentation of bra size or cup size. Premastectomy bra size was obtained from 76 patients through phone calls or emails. The remaining 159 patients either refused to participate, did not respond, or were not contacted. To minimize confounding by postoperative swelling, patients whose postmastectomy bra size documentation occurred within 3 months after mastectomy were excluded from analysis. For the 211 patients with known premastectomy bra cup and band sizes, chart reviews were performed to acquire the weight of breast tissue removed by mastectomy. Upon review of pathology reports, 2 patients did not have documented specimen weights, resulting in 209 patients for the final analysis. Each of 209 patients was assigned a sister bra size group according to Table 1. Demographic information was acquired manually by chart review.

**Table 1.** Sister Bra Size Theory

	Band size							
Sister bra size group	30	32	34	36	38	40	42	44
1	30AA							
2	30A	32AA						
3	30B	32A	34AA					
4	30C	32B	34A	36AA				
5	30D	32C	34B	36A	38AA			
6	30DD/E	32D	34C	36B	38A	40AA		
7	30DDD/F	32DD/E	34D	36C	38B	40A	42AA	
8	30G	32DDD/F	34DD/E	36D	38C	40B	42A	44AA
9	30H	32G	34DDD/F	36DD/E	38D	40C	42B	44A
10	30I	32H	34G	36DDD/F	38DD/E	40D	42C	44B
11	30J	32I	34H	36G	38DDD/F	40DD/E	42D	44C
12		32J	34I	36H	38G	40DDD/F	42DD/E	44D
13			34J	36I	38H	40G	42DDD/F	44DD/E
14				36J	38I	40H	42G	44DDD/F
15					38J	40I	42H	44G
16						40J	42I	44H
17							42J	44I
18								44J

### Pearson's and Spearman's Correlation Analyses

Statistical analysis was performed using each breast as an individual data point. Of the 209, 186 patients underwent bilateral mastectomy and 23 underwent unilateral mastectomy, yielding 395 breasts. All analyses were performed using Microsoft Excel. Both Spearman's and Pearson's correlation coefficients were calculated between mastectomy specimen weight and sister bra size group, BMI, BMI group, band size alone, cup size alone, and age. To calculate correlation coefficients for bra cup size and the weight of breast tissue removed during mastectomy, bra cup sizes were first converted to numerical values (eg, AA = 1, A = 2, B = 3, and so on) based on a sister bra size conversion chart (Table 1). The other variables were already numeric and were not transformed before calculating correlation coefficients. Age was included as a control variable because no correlation with breast weight was expected. A Fisher's *r* to *z* transformation was performed to normalize the *r* values for Pearson analysis. Correlations were then assigned as a “positive correlation” or “negative correlation” based on the sign of the *r* value. Linear correlations were categorized as perfect (1), strong (0.71-0.99), moderate (0.3-0.7), weak (0.1-0.29), or no significant correlation (0).

### Multivariate Linear Regression Analysis

Multivariate linear regression was performed using Python (v3.11; statsmodels) to assess the independent relationship of each variable with breast weight. Sister size, cup size, band size, and BMI were included as covariates. *R*^2^, adjusted *R*^2^, and *F*-statistic were utilized to evaluate model performance. Statistical significance was defined as *P* < .05.

## RESULTS

### Demographic Information

The study's demographic characteristics are outlined in Table 2. There was a total of 209 patients included in the study, with no male or nonbinary participants (100% female). The median age at the time of surgery was 45 years, with an age range of 23 to 78 years. The racial demographics of the participants included categories for Asian (*n* = 22, 11%), Black or African American (*n* = 6, 3%), Middle Eastern or North African (*n* = 2, 1%), Native Hawaiian or Other Pacific Islander (*n* = 2, 1%), White or Caucasian (*n* = 85, 41%), multiple races (*n* = 10, 5%), and other race (*n* = 38, 18%), or unknown (*n* = 44, 21%). Thirty-four (16%) of the patients identified as Hispanic or Latino in ethnicity.

**Table 2.** Demographic Information and Mastectomy Characteristics

Category	Characteristic	*n* (%)
Sex		
	Female	209 (100)
	Male	0 (0)
BMI at time of surgery Mean (range) = 26 (16-45)		
	16-24	94 (45)
	25-29	56 (27)
	30-35	43 (21)
	36+	11 (5)
	Unspecified	5 (2)
Age at time of surgery (years) Mean (range) = 45 (23-78)		
	18-24	3 (1)
	25-34	28 (13)
	35-44	78 (37)
	45-54	60 (29)
	55-64	30 (14)
	65+	7 (3)
	Unspecified	3 (1)
Ethnicity		
	Hispanic or Latino	34 (16)
	Non-Hispanic or Latino	144 (69)
	Unknown	31 (15)
Race		
	Asian	22 (11)
	Black or African American	6 (3)
	Middle Eastern or North African	2 (1)
	Native Hawaiian or Other Pacific Islander	2 (1)
	White or Caucasian	85 (41)
	Multiple races	10 (5)
	Other race	38 (18)
	Unknown	44 (21)
Mastectomy type		
	Simple (total)	108 (27)
	Nipple sparing	107 (27)
	Skin sparing	180 (46)
Mastectomy laterality		
	Bilateral	186 (89)
	Unilateral	23 (11)

*n* = number of patients.

### Mastectomy Type

Of the 209 patients, 108 patients (27%) underwent a simple (total) mastectomy and 107 patients (27%) underwent a nipple-sparing mastectomy. Skin-sparing mastectomy was the most common mastectomy type, with 180 patients (46%). The majority of patients underwent bilateral mastectomy (*n* = 186, 89%). Twenty-three patients (11%) underwent unilateral mastectomy. The total number of individual breasts that underwent mastectomy was 395.

### Sister Bra Size Groups

Each of the 395 breasts was assigned to a sister bra size group according to their premastectomy bra size and Table 1. The sister bra size group assignments ranged from 3 to 13, with no patients on the smaller end of the scale (Sister bra size groups 1 and 2) and no patients on the larger end of the scale (Sister bra size groups 14+). Sister bra size groups 3, 11, 12, and 13 all had an *n* of <10 breasts, with 2, 2, 7, and 5 breasts, respectively. Sister bra size groups 4, 9, and 10 had a study group population between 30 and 40 breasts, with 38, 38, and 34 breasts, respectively. Sister bra size groups 7 and 8 had similar numbers of breasts, with 58 and 60 breasts, respectively. Groups 5 and 6 were the most common sister bra size groups, with 73 and 78 breasts, respectively.

### Testing for Predictors of Breast Tissue Weight

**Figure 2.**
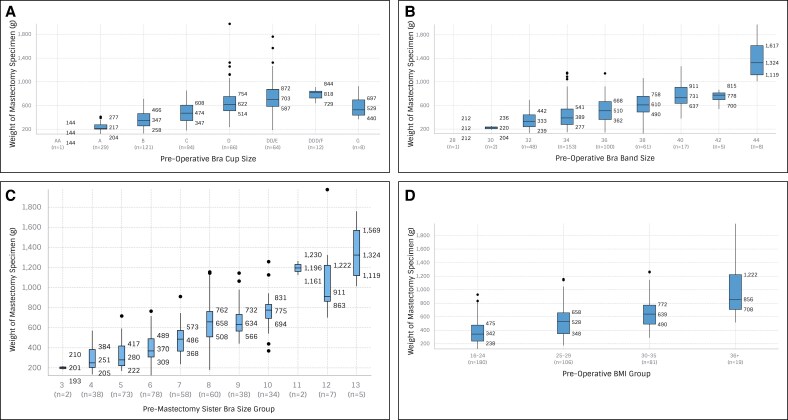
Predictors of mastectomy specimen weight. (A) Premastectomy sister bra size group vs mastectomy specimen weight (g) (*n* = 395 breasts). (B) Premastectomy bra band size vs mastectomy specimen weight (g) (*n* = 395 breasts). (C) Premastectomy bra cup size vs mastectomy specimen weight (g) (*n* = 395 breasts). (D) Premastectomy BMI group vs mastectomy specimen weight (g) (*n* = 386 breasts).

Figure 2 illustrates with box-and-whisker plots the relationship between preoperative sister bra size groups and the weight of the mastectomy specimens (in grams) among 395 individual breasts. Upon visualization of the box-and-whisker plots, there is a general trend in which larger preoperative bra size groups correspond to greater mean weights of the removed mastectomy specimens. Additionally, the median weight of the mastectomy specimens increases with higher bra size groups. For instance, patients in Group 4 had a median specimen weight of 287 g, with a range of 135 to 570 g. Conversely, patients in the largest bra size group, Group 13, had a median specimen weight of 1327 g, with a range extending from 1013 to 1759 g. As hypothesized, sister bra size demonstrated a strong correlation with breast tissue weight (*ρ* = 0.76, *r* = 0.77). BMI (*ρ* = 0.61, *r* = 0.61), cup size (*ρ* = 0.67, *r* = 0.60), and band size (*ρ* = 0.48, *r* = 0.65) all showed moderate correlations, although significant (Table 3). As expected, age did not have a correlation with breast tissue weight (*ρ* = 0.02, *r* = 0.07).

**Table 3.** Predictors of Mastectomy Specimen Weight

Variable	Spearman's *ρ*	Spearman *P*-value	Pearson's *r*	Fisher’s *z*-score	Pearson *P*-value	*r* ^2^ (Pearson)	Correlation type
Sister bra size (*n* = 395)	0.76	<.001	0.77	1.01	<.001	0.59	Strong, positive
BMI (*n* = 386)	0.61	<.001	0.61	0.7	<.001	0.37	Moderate, positive
Bra cup size (*n* = 395)	0.67	<.001	0.60	0.69	<.001	0.36	Moderate, positive
BMI group (*n* = 386)	0.56	<.001	0.59	0.68	<.001	0.35	Moderate, positive
Bra band size (*n* = 395)	0.48	<.001	0.57	0.65	<.001	0.32	Moderate, positive
Age (*n* = 395)	0.02	.735	0.07	0.07	.215	0.01	No significant correlation

### Multivariate Linear Regression Analysis

The results of our multivariate linear regression analysis are illustrated in Table 4. Sister size (*P* = .016) and BMI (*P* < .001) were found to be independently associated with specimen weight, whereas cup size (*P* = .997) and band size (*P* = .775) were not significant predictors. The final model was statistically significant (*P* < .001) and explained ∼63% of the variation in breast weight, indicating a strong fit and highlighting the clinical utility of using sister size in surgical planning.

**Table 4.** Multivariate Linear Regression Model Predicting Breast Weight

Variable	Coefficient *β*	Standard error	*t*-Value	*P*-value	95% CI (lower, upper)
Intercept	−245	536	−0.46	.648	−1299, 809.0
Sister size group	83.8	34.5	2.43	.016	15.97, 151.6
BMI	13.7	2.08	6.59	<.001	9.600, 17.78
Cup size	0.14	32.4	0.00	.997	−63.61, 63.88
Band size	−5.15	18.0	−0.29	.775	−40.59, 30.30

Model *R*^2^ = 0.628; adjusted *R*^2^ = 0.624; *n* = 386; *F*-statistic = 160.6; *P* (model) < .0001.

## DISCUSSION

**Figure 3.**
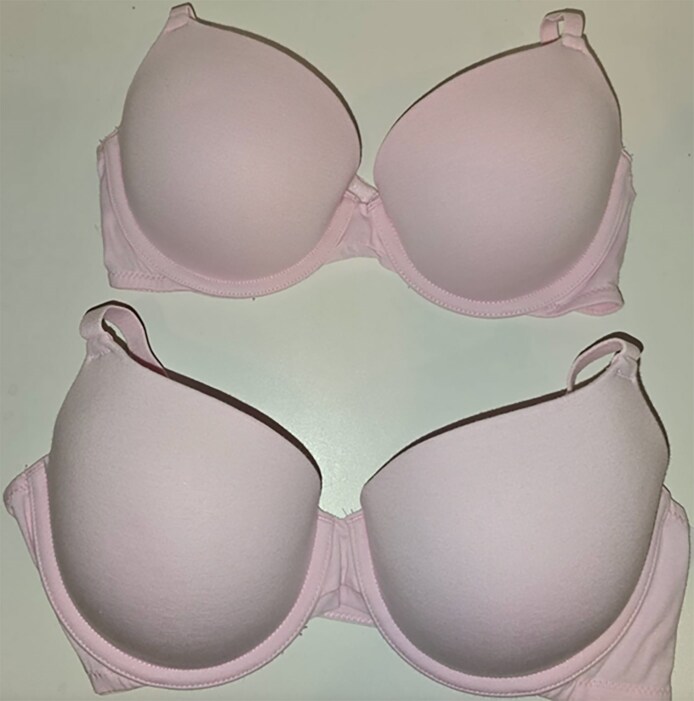
Comparison of 2 bras from the same manufacturer with identical cup sizes but differing band and sister sizes. Top: 34B (sister size 5). Bottom: 38B (sister size 7).

The authors of this study aim to explore the relationship between premastectomy bra sizes and the weight of breast tissue removed during mastectomy. The findings demonstrate a significant, strong positive correlation between preoperative bra size and the weight of the mastectomy specimen. As preoperative bra size increased, both the mean and median weights of the breast tissue removed increased, which was consistent across our sample of 395 individual breasts (Table 5). It is expected that a larger bra size corresponds to a heavier breast weight. However, it is important to include both band and cup size rather than solely cup size. Our dataset shows that a patient with a C cup can be in Sister bra size groups 5 to 10, with Group 5 having an average individual breast weight of 325 g and Group 10 having an average individual breast weight of more than twice that weight (771 g). In other words, a patient with a 32C bra (Sister bra group 5) has significantly smaller breast sizes than a patient with a 42C bra (Sister bra group 10), although they are both C cups. Figure 3 illustrates this discrepancy, presenting 2 bras made by the same company that are both B cups yet vastly different in size.

**Table 5.** Mastectomy Specimen Weights by Preoperative Sister Bra Size Group

BMI group			Weight of breast tissue removed (g)					
	Sister bra size group	*n* (no. of breasts)	Average (g)	Median (g)	Quartile 1	Quartile 3	Minimum (g)	Maximum (g)
All patients (*n* = 395)	3	2	201	201	193	210	184	218
	4	38	287	251	205	384	135	570
	5	73	325	280	222	417	170	716
	6	78	396	370	309	489	126	764
	7	58	488	486	368	573	237	910
	8	60	660	658	508	762	180	1155
	9	38	659	634	566	732	440	1144
	10	34	771	775	694	831	370	1258
	11	2	1196	1196	1161	1230	1126	1265
	12	7	1108	911	863	1222	700	1975
	13	5	1357	1324	1119	1569	1013	1759
	All	395	511	478	311	663	126	1975
16-24 (Group 1, *n* = 190)	3	2	201	201	193	210	184	218
	4	38	259	231	201	295	135	442
	5	48	293	256	217	350	170	530
	6	46	349	339	253	452	126	530
	7	22	443	468	332	507	261	623
	8	16	537	580	427	658	251	745
	9	12	582	598	490	654	440	705
	10	6	789	786	743	830	666	925
	11-13	0	—	—	—	—	—	—
	All	190	378	342	238	475	126	925
25-29 (Group 2, *n* = 106)	3	0	—	—	—	—	—	—
	4	8	358	345	247	450	173	570
	5	19	378	363	275	457	204	716
	6	14	386	334	314	428	273	665
	7	26	523	561	422	601	237	910
	8	21	698	687	567	800	180	1155
	9	10	619	593	533	729	458	795
	10	8	707	805	587	853	370	881
	11-13	0	—	—	—	—	—	—
	All	106	524	528	348	658	173	1155
30-35 (Group 3, *n* = 81)	3	0	—	—	—	—	—	—
	4	2	400	400	391	409	382	418
	5	4	531	513	501	552	489	591
	6	15	522	513	425	587	330	764
	7	10	495	485	379	601	287	744
	8	21	689	679	557	833	378	1144
	9	10	742	703	606	772	569	1064
	10	16	810	775	697	818	636	1258
	11	2	1196	1196	1161	1230	1126	1265
	12	0	—	—	—	—	—	—
	13	1	1013	1013	1013	1013	1013	1013
	All	81	671	639	490	772	287	1265
36+ (Group 4, *n* = 19)	3-5	0	—	—	—	—	—	—
	6	1	716	716	716	716	716	716
	7-8	0	—	—	—	—	—	—
	9	4	615	607	559	664	515	731
	10	3	717	787	664	807	540	826
	11	0	—	—	—	—	—	—
	12	7	1108	911	863	1222	700	1975
	13	4	1143	1447	1273	1617	1119	1759
	All	19	992	856	708	1222	515	1975

Sister bra size group, with a Spearman's coefficient of 0.76, demonstrated a positive strong correlation with breast tissue weight. The strong and consistent correlation between sister size and breast weight across both Pearson (*r* = 0.77) and Spearman (*ρ* = 0.76) supports its potential as a predictive metric. The minimal difference between the 2 coefficients suggests a stable relationship regardless of linearity assumptions. BMI, cup size, and band size all demonstrated moderate correlations. Band size and cup size both demonstrated greater variability between correlation types, supporting our hypothesis that they are less reliable metrics. Multivariate linear regression analysis confirmed that sister size and BMI are independently associated with breast weight, whereas cup and band size alone are not significant predictors.

The strong correlation between sister size and breast weight is particularly important for presurgical planning because it can help surgeons in anticipating the extent of tissue removal necessary, as well as in making decisions about the appropriate surgical approach. Additionally, the patient can know more of what to expect from their body postoperatively. For example, patients with larger sister bra sizes usually require more extensive tissue removal, which could influence decisions about the type of mastectomy performed and whether immediate reconstruction is feasible. Our results suggest that employing the Sister Bra Size Theory may enhance the accuracy of documentation for breast surgery candidates. Routinely including band size and sister bra size group in a breast surgery candidate's medical record documentation by incorporating “band size,” “cup size,” and “sister bra size group” fields in note templates, for example, can facilitate future clinical research studies.

A potential utilization of these findings is the formation of a calculator that can be utilized to determine the mass of breast tissue required to be removed to achieve a desired postreduction size. The calculator could be based on a mathematical model that uses sister bra size groups and corresponding breast tissue weights of mastectomy patients and the preoperative sister bra size groups of patients seeking breast reduction. Additionally, our results may assist in postmastectomy implant selection. Given the correlation between mastectomy specimen weight and breast volume, and the strong correlation with sister bra size group, patient-reported bra size may potentially serve as a practical surrogate for estimating implant volume.^9^ A reliable estimate of premastectomy breast size may be especially helpful in unilateral mastectomy cases, where implant selection aims to achieve symmetry with the contralateral breast. Future research could evaluate the relationship between sister bra size and final implant size, as well as the relationship between premastectomy breast width and mastectomy weight, to aid in implant selection.

This study has some limitations to be considered when interpreting the results. The use of bra size as a proxy for breast volume has some inherent inaccuracies, because bra sizing is not standardized between manufacturers. We relied solely on patient-reported bra sizes rather than measuring bra sizes ourselves, given that, in clinical practice, it is common for surgeons to document patient-reported bra sizes. Recognizing this limitation, we have initiated a subsequent study aimed at developing a calculated sister size index that utilizes standard clinical breast measurements. This model is trained on validated sister size data from the current cohort and incorporates prereduction surgery candidates to ensure representation of larger bra sizes. The resulting continuous sister size index is intended to further enhance preoperative decision making and surgical planning. Additionally, bra size does not account for breast density or shape, which may also influence the weight of the tissue removed.

Future studies could benefit from the use of more precise measurements of breast volume, such as 3-dimensional imaging or volumetric analysis from MRI, to further uncover the relationship between bra size and removed specimen weight.

## CONCLUSIONS

In this study, we set out to test the Sister Bra Size Theory—a concept that refers to the idea that several different bra sizes can fit the same breast depending on the combination of band size and cup size. This theory suggests that the volume of the cup size changes with the band size of the bra, and the equivalent sizes are known as “sister sizes.” Our analysis of the premastectomy bra sizes and mastectomy specimen weights of 395 breasts confirms that the sister bra size group is a strong predictor of breast size. Band and cup sizes alone are only moderate predictors of breast weight. Finally, we statistically validated the Sister Bra Size Theory and demonstrated that it has a strong correlation with breast weight regardless of variation in bra brand. Surgeons will benefit from documenting a patient's sister bra size rather than cup or band size alone. Our findings can be utilized in the future in the creation of a calculator that uses sister bra size groups and corresponding breast tissue weights of mastectomy patients to predict the mass of breast tissue required to be removed to achieve a desired postreduction size. Future research could include incorporation of a breast's adipose tissue to fibrous tissue composition to assess the relationship of breast density to bra size.

## References

[1] Ehrenstein V, Kharrazi H, Lehmann H, et al Obtaining data from electronic health records. In: Gliklich RE, Leavy MB, Dreyer NA, eds. Tools and Technologies for Registry Interoperability, Registries for Evaluating Patient Outcomes: A User's Guide, 3rd ed. Agency for Healthcare Research and Quality; 2019:52–59. 31891455

[2] Sauer CM, Chen LC, Hyland SL, Girbes A, Elbers P, Celi LA. Leveraging electronic health records for data science: common pitfalls and how to avoid them. Lancet Digit Health. 2022;4:e893–e898. doi: 10.1016/S2589-7500(22)00154-6 36154811

[3] Siegel RL, Giaquinto AN, Jemal A. Cancer statistics, 2024. CA Cancer J Clin. 2024;74:1–114. doi: 10.3322/caac.21882 38230766

[4] Arzanova E, Mayrovitz HN. The epidemiology of breast cancer. In: Mayrovitz HN, ed. Breast Cancer. Exon Publications; 2022:1–19. 36122161

[5] Malphrus EL, Desai A, Weiss ES, Couto JA, Broach R, Butler PD. Understanding public perception of bra size. J Plast Reconstr Aesthet Surg. 2022;75:4197–4201. doi: 10.1016/jbjps.2022.08.038 36180339

[6] Bustyresources Wiki . Sister size. 2025. Accessed February 14, 2025. https://bustyresources.fandom.com/wiki/Sister_size

[7] Hepworth A . “Sister Size” for Bras: What Are They and Do They Work? PureWow; 2019. Accessed February 14, 2025. https://www.purewow.com/fashion/sister-size-bras

[8] Wang W, Ferrari D, Haddon-Hill G, et al Electronic health records as source of research data. In: Colliot O, ed. Machine Learning for Brain Disorders. Humana; 2023:331–350. 37988545

[9] Wazir U, El Hage Chehade H, Choy C, Kasem A, Mokbel K. A study of the relation between mastectomy specimen weight and volume with implant size in oncoplastic reconstruction. In Vivo. 2019;33:125–132. doi: 10.21873/invivo.11448 30587612 PMC6364091

